# Calmodulin Contributes to Lipolysis and Inflammatory Responses in Clinical Ketosis Cows through the TLR4/IKK/NF-κB Pathway

**DOI:** 10.3390/ani14111678

**Published:** 2024-06-04

**Authors:** Jinshui Chang, Zhijie Wang, Yu Hao, Yuxi Song, Cheng Xia

**Affiliations:** 1 College of Animal Science and Veterinary Medicine, Heilongjiang Bayi Agricultural University, Daqing 163319, China; cjs19980225@163.com (J.C.); 18753930939@163.com (Y.H.); syxalz@163.com (Y.S.); 2 College of Veterinary Medicine, Southwest University, Chongqing 400715, China; wangzhijie97@163.com; 3 Key Laboratory of Bovine Disease Control in Northeast China, Ministry of Agriculture and Rural Affairs, Daqing 163319, China

**Keywords:** calmodulin, ketosis, lipolysis, inflammation, adipocytes, TLR4/IKK/NF-κB

## Abstract

**Simple Summary:**

Clinical ketosis is a dangerous disease in the dairy industry, as it affects milk production in cows. We imply that calmodulin is abundantly expressed in adipocytes during lipolysis and inflammatory responses. Thus, reducing calmodulin levels could help control the inflammatory response in the adipose tissue of dairy cows during the development of clinical ketosis.

**Abstract:**

Clinical ketosis is a detrimental metabolic disease in dairy cows, often accompanied by severe lipolysis and inflammation in adipose tissue. Our previous study suggested a 2.401-fold upregulation in the calmodulin (CaM) level in the adipose tissue of cows with clinical ketosis. Therefore, we hypothesized that CaM may regulate lipolysis and inflammatory responses in cows with clinical ketosis. To verify the hypothesis, we conducted a thorough veterinary assessment of clinical symptoms and serum β-hydroxybutyrate (BHB) concentration. Subsequently, we collected subcutaneous adipose tissue samples from six healthy and six clinically ketotic *Holstein* cows at 17 ± 4 days postpartum. Commercial kits were used to test the abundance of BHB, non-esterified fatty acid (NEFA), the liver function index (LFI), interleukin-6 (IL-6), IL-1β, and tumor necrosis factor-α (TNF-α). We found that cows with clinical ketosis exhibited higher levels of BHB, NEFA, LFI, IL-6, IL-1β, TNF-α, and lower glucose levels than healthy cows. Furthermore, the abundance of CaM, toll-like receptor 4 (TLR4), inhibitor of nuclear factor κB kinase subunit β (IKK), phosphorylated nuclear factor κB p65/nuclear factor κB p65 (p-NF-κB p65/NF-κB p65), adipose triacylglycerol lipase (ATGL), and phosphorylated hormone-sensitive lipase/hormone-sensitive lipase (p-HSL/HSL) was increased, while that of perilipin-1 (PLIN1) was decreased in the adipose tissue of cows with clinical ketosis. To investigate the mechanism underlying the responses, we isolated the primary bovine adipocytes from the adipose tissue of healthy cows and induced the inflammatory response mediated by TLR4/IKK/NF-κB p65 with lipopolysaccharide (LPS). Additionally, we treated the primary bovine adipocytes with CaM overexpression adenovirus and CaM small interfering RNA. In vitro, LPS upregulated the abundance of TLR4, IKK, p-NF-κB p65, ATGL, p-HSL/HSL, and CaM and downregulated PLIN1. Furthermore, CaM silencing downregulated the abundance of LPS-activated p-HSL/HSL, TLR4, IKK, and p-NF-κB p65 and upregulated PLIN1 in bovine adipocytes, except for ATGL. However, CaM overexpression upregulated the abundance of LPS-activated p-HSL/HSL, TLR4, IKK, and p-NF-κB p65 and downregulated PLIN1 expression in bovine adipocytes. These data suggest that CaM promotes lipolysis in adipocytes through HSL and PINL1 while activating the TLR4/IKK/NF-κB inflammatory pathway to stimulate an inflammatory response. There is a positive feedback loop between CaM, lipolysis, and inflammation. Inhibiting CaM may act as an adaptive mechanism to alleviate metabolic dysregulation in adipose tissue, thereby relieving lipolysis and inflammatory responses.

## 1. Introduction

In 1995, the transition period in cows was defined as three weeks before and after calving [1]. Cows undergo significant metabolic and endocrine changes during this period in preparation for parturition and lactation. Ketosis is a major metabolic disorder of high-yielding dairy cows during the transition period, characterized by elevated blood concentrations of β-hydroxybutyrate (BHB) and non-esterified fatty acid (NEFA) resulting from intense lipolysis [2,3,4]. Adipose triacylglycerol lipase (ATGL), hormone-sensitive lipase (HSL), and perilipin 1 (PLIN1) are crucial in lipolysis in dairy cows. Additionally, ATGL, HSL, and monoacylglycerol lipase hydrolyze triacylglycerol to diacylglycerol and monoacylglycerol glycerol, respectively, and fatty acids are released in this process [5,6]. PLIN1 usually covers the surface of lipid droplets to prevent excessive lipolysis. Lipolysis is initiated when comparative gene identification-58 on PLIN1 is transposed to ATGL, exposing lipid droplets [6,7]. Drastic lipolysis implies a large amount of NEFA production. The liver showed the characteristics of liver function injury, such as increased positive acute phase reactive protein, aspartate aminotransferase (AST) and alanine transaminase (ALT) levels, and a decreased liver function index (LFI) [8]. However, NEFA can act on toll-like receptor 4 (TLR4), activate nuclear factor κB (NF-κB), and promote the secretion of tumor necrosis factor-α (TNF-α), interleukin-1β (IL-1β), interleukin-6 (IL-6), and other inflammatory factors [9,10].

Calmodulin (CaM) is a highly conserved signal regulator protein that regulates cell division, development, proliferation, autophagy, and lipolysis by binding to different target proteins [11,12,13]. Furthermore, CaM is associated with inflammation and lipolysis in many tissues [14,15,16]. Calmodulin kinase II (CAMKII) enhances lipolysis of fat and TNFα-induced inflammation [12]. In a study of post-traumatic osteoarthritis in mice, inhibition of CAMKII attenuated chondrocyte inflammation [17]. In another study, imperatorin ameliorates mast cell-mediated allergic airway inflammation by inhibiting Mas-related G protein-coupled receptor-X2 and CaMKII/extracellular regulated protein kinases (ERK) signaling pathways [18]. There are no reports on the relationship between CaM and the pathogenesis of ketosis in dairy cows. Our previous proteomic study found that CaM expression was higher in the adipose tissue of cows with ketosis with a difference of 2.401 times [19], suggesting a link between CaM, lipolysis, and the inflammatory response.

This study assessed the association between in vitro and in vivo CaM, lipolysis, and inflammation. We found that cows with ketosis exhibited enhanced lipolysis, an inflammatory response, and adipose tissue CaM expression. Notably, our results demonstrated that CaM in the primary adipocytes of dairy cows could activate lipolysis and an inflammatory response. Furthermore, inflammatory cytokines produced by an inflammatory response could promote CaM expression and the activation of lipolysis in adipose tissue. This study provides new insights into regulating lipid metabolism and the inflammation mechanism in clinical ketosis cows.

## 2. Materials and Methods

### 2.1. Ethics Statement

The experiment was conducted on a large intensive cattle farm with 6000 *Holstein* cows located in the central region of Heilongjiang Province, China. All animal procedures were performed in accordance with the Guidelines for the Care and Use of Experimental Animals at Heilongjiang Bayi Agricultural University (Daqing, China) (DWKJXY2023064, approval date: 1 January 2023). All methods were reported in accordance with ARRIVE guidelines 2.0 [20].

### 2.2. Animals

This study included 150 lactating *Holstein* cows. These cows exhibited similar parity (median = 3, range = 2–4) and milk production duration (median = 17 days, range = 12–21 days). The study was conducted between January and February 2023 at a cow dairy farm in Daqing City (Heilongjiang, China), which has a free-stall housing system with a herd of 6000 cows. The cows were tested to identify those with ketosis and healthy animals. Thorough examinations were conducted on all cows to ensure the absence of any additional complications, including hypocalcemia or mastitis. Body condition was assessed by the same herd veterinarian using a 5-point scale (1 = emaciated to 5 = obese) [21]. Based on clinical symptoms and serum BHB concentration, six cows with clinical ketosis with serum BHB concentrations > 3.0 mmol/L were randomly selected as the clinical ketosis group (CK), and six cows with serum BHB concentrations < 1.20 mmol/L were randomly selected as the healthy control group (CON) [22]. Table 1 shows the characteristics of milk production, dry matter intake (DMI), body weight (BW), serum glucose, BHB, and NEFA concentrations of selected control and clinical ketosis cows. The total mixed ration of tested dairy cows met dairy cows’ requirements. All cows were fed the same diet that complied with the NRC (2001) and Chinese feeding standards during the experiment. Supplementary Table S1 illustrates the diet composition of the cows during the experimental period.

### 2.3. Collection of Blood

Blood samples (CON, *n* = 6, CK, *n* = 6) were collected on three consecutive days at the same hour daily via jugular venipuncture without anticoagulant. The first blood sample was collected during clinical examination, and the other three samples were collected every 24 h. The blood samples were collected between 0600 and 0800 h before feeding, and the serum was separated after centrifugation at 3500× *g* for 10 min at 4 °C and stored at −80 °C until analysis.

### 2.4. Biomarkers

Serum AST (Nanjing Jiancheng Bioengineering Institute, Nanjing, China, cat. no. F006-1-1), ALT (Nanjing Jiancheng Bioengineering Institute, Nanjing, China, cat. no. C009-3-1), glucose (Nanjing Jiancheng Bioengineering Institute, Nanjing, China, cat. no. F006-1-1), NEFA (Nanjing Jiancheng Bioengineering Institute, Nanjing, China, cat. no. A042-1-1), and BHB (Randox Laboratories, Crumlin, UK, cat. no. RB1008) were measured using the Mindray BS-830 autoanalyzer (Mindray Laboratory Measurement, Shenzhen, China) and commercial kits. The Infinite M200 PRO raster multifunctional microplate reader (Tecan Company, Männedorf, Switzerland) and commercial kits (XIN FAN BIOLOGY, Shenzhen, China) were used to detect IL-6 (cat. no. XFH10555), TNF-α (cat. no. XFH13476), and IL-1β (cat. no. XFH10519). Furthermore, the detection and calculation of the LFI were conducted according to our previous study [23].

### 2.5. Adipose Tissue Collection and Processing

Subcutaneous adipose tissue samples were collected from the tail of the cows on the same day at 17 (±4) days postpartum by an experienced veterinarian using the methods previously described (CON, *n* = 6, CK, *n* = 6) [24]. Before tissue collection, the pelage in the tail-head region and one side of the tail-head were meticulously cleansed using surgical soap. Local anesthesia was administered within the area encompassing the ischium and tailbone. A scalpel incision measuring approximately 6–8 cm was performed, and the skin was gently pulled back using sterile forceps and hemostats to expose the underlying tissue. Adipose tissue samples weighing between 1 and 2 g were meticulously collected using sterile scalpel blades and forceps. Following sampling, compression hemostasis was carefully applied with sterile gauze to prevent any external bleeding. The sampling incision was closed using 8 to 12 surgical staples for optimal wound closure. Subsequently, adipose tissue samples were thoroughly washed with a sterile phosphate-buffered saline (PBS) solution. For total protein extraction purposes, adipose tissue samples were accurately weighed and stored in cryotubes before being promptly placed into liquid nitrogen for preservation. Another portion of tissue was preserved for immunohistochemistry using tissue fixative (Servicebio, Wuhan, China, Cat: G1119).

### 2.6. Immunohistochemistry Technology

According to Wang’s method [25], fresh subcutaneous adipose tissue was cut into 1.5 cm × 1.5 cm × 0.3 cm and fixed in adipose tissue-specific fixative (Servicebio, Wuhan, China). The adipose tissue was dehydrated using 85%, 95%, and 100% ethanol. Paraffin was melted in an incubator at 58–60 °C before embedding. After the paraffin was sufficiently cooled, the sections were made. The slices were incubated at 37 °C overnight, followed by deparaffinization. Subsequently, the sections were placed in xylene solution treated with ethanol at 100%, 90%, 80%, and 70% and gently rinsed with PBS. The dewaxing tissue was immersed in ethylenediamine tetraacetic acid (EDTA) antigen repair buffer (Servicebio, Wuhan, China, Cat: G1206), heated for 5 min at 98 °C, cooled, and placed in PBS buffer (PH = 7.4). The antigen repair was completed by washing the tissue with PBS buffer (PH = 7.4) thrice for 5 min. The slides were added into the working solution of the primary antibody drop (Rabbit CaM antibody, 1:1000, Abcam Cambridge, UK, Cat: ab45689), incubated at 37 °C for 1 h, and rinsed with PBS. Subsequently, the slides were added into the working solution of the secondary antibody drop (Goat Anti-Rabbit IgG, 1:200, Servicebio, Wuhan, China, Cat: G1213), incubated at 37 °C for 30 min, and rinsed with PBS. The sectioned tissues were counterstained using hematoxylin, dehydrated with 70%, 80%, and 90% absolute ethanol, and sealed with neutral gum. The image information was observed and collected using a microscope (Carl Zeiss, Inc. Thornwood, NY, USA).

### 2.7. Isolation of Bovine Preadipocytes

Preadipocytes were isolated using the method published in previous studies [26]. Three healthy female one-day-old *Holstein* calves (40–50 kg) were selected and slaughtered after anesthesia with thiamylal sodium (50 mg/kg). Adipose tissues of the greater omentum and mesentery of the calves were collected using aseptic procedures and washed thrice with PBS containing penicillin (2500 U/mL) and streptomycin (2500 mg/mL) (Solarbio, Beijing, China). The collected adipose tissues were loaded into a sterile beaker. The blood vessels and fascia in the adipose tissue were removed, and the adipose tissue (1 g) was enzymatically digested using a collagenase type I digestion solution (final concentration 1 mg/mL; Sigma-Aldrich, St. Louis, MO, USA) and digested in a 37 °C water bath with shaking table for 60 min. The digested tissue was filtered sequentially through 20-mesh and 200-mesh screens, and the filtrate was centrifuged at 1000× *g* for 10 min. The supernatant was discarded, and the precipitate was resuspended by adding the DMEM/F12 medium (HyClone, Logan, UT, USA) into a 10% fetal bovine serum (HyClone, Logan, UT, USA) and re-centrifuged at 1000× *g* for 10 min. After resuspension with basic culture medium (BCM) (10% fetal bovine serum and 1% gentamycin) medium, the cells were inoculated in cell culture flasks at 37 °C for 24 h under 5% CO_2_ in a cell incubator. The medium was replaced to remove the remaining tissue and non-adherent cells. The medium was changed every 48 h.

### 2.8. Cell Culture and Processing

According to the method previously published by Xu [27], to induce preadipocyte differentiation, we used the BCM containing 0.5 mM 3-isobutyl-1-methylxanthine (Sigma-Aldrich), 1 μM dexamethasone (Sigma-Aldrich), and 1 μg/mL insulin (Sigma-Aldrich). The cells were cultured at 37 °C in 5% CO_2_ for two days and replaced with BCM containing 1 μg/mL insulin (Sigma-Aldrich) to induce differentiation further. The medium was refreshed every 2 days until visible lipid droplets were observed in the cell, signifying the completion of cellular differentiation. This duration spanned approximately 10 days [28,29].

After adipocyte differentiation, the medium was changed to a BCM medium containing 4 μg/mL LPS (Solarbio, Beijing, China) for 3 h. In transfection experiments, the adipocytes were treated with CaM small interfering RNA (si-CaM) and CaM overexpression adenovirus (OC-CaM) for 48 h. The detailed grouping is described in the figure legends. The overexpression adenovirus vector (OC-CaM) was constructed using Hanbio (Shanghai, China). Similarly, siRNAs were designed and synthesized using Hanbio (Shanghai, China) based on bovine CaM mRNA sequences. The siRNA sequences are illustrated as follows: GGUGAUGGCACCAUCACAATT, Antisense strand: UUGUGAUGGUGCCAUCACCTT.

### 2.9. Western Blotting

Total protein from adipose tissue and adipocytes was extracted using a protein extraction kit (Beyotime Biotechnology, Shanghai, China) according to the manufacturer’s instructions. For adipose tissue, 70 mg of adipose tissue was taken, RIPA (Roche, Shanghai, China) tissue lysate was added, and adipose tissue was subsequently added to a grinder. The ground tissue was centrifuged using a centrifuge (3–16 KL, SIGMA, Osterode, Germany) at 12,000× *g* for 10 min at 4 °C. Protein extraction reagent was added to the centrifuged supernatant, thoroughly mixed, and re-centrifuged at 12,000× *g* at 4 °C for 10 min to obtain total adipose tissue protein. For adipocytes, cells were collected from 0.25% trypsin-EDTA (T1320, Solarbio, Beijing, China) and digested by centrifugation at room temperature, 1500× *g* for 5 min. After discarding the supernatant, the centrifugation steps described above were repeated after the resuspension of the cells using PBS. After discarding the supernatant, the cells were resuspended in precooled lysis buffer and centrifuged at 12,000× *g* for 10 min at 4 °C to obtain total protein from the adipocytes. Total protein in adipose tissue and adipocytes was determined using the BCA method. Typically, 30 μg of protein from each sample was separated using sodium dodecyl sulfate-polyacrylamide gel electrophoresis (SDS-PAGE) with known molecular weight markers, and the proteins were transferred to a 0.45-μm PVDF membrane. The PVDFs were incubated with primary antibodies anti-β-actin (1:1000, Proteintech Group, Chicago, IL, USA), anti-Toll-like receptor 4 (TLR4, 1:1000, Proteintech Group, Chicago, IL, USA), anti-IKK (1:1000, Proteintech Group, Chicago, IL, USA), anti-NF-κB (1:4000, Proteintech Group, Chicago, IL, USA), anti-p-NF-κB (1:2000, Cell Signaling Technology, Boston, MA, USA), anti-ATGL (ATGL, 1:1000, Proteintech Group, Chicago, IL, USA), and anti-hormone-sensitive lipase (HSL, 1:2000, Proteintech Group, Chicago, IL, USA). Anti-phosphorylated hormone-sensitive lipase (p-HSL, 1:1000, ABclonal, Boston, MA, USA), anti-perilipin-1 (PLIN1, 1:2000, ABclonal, Boston, MA, USA), and anti-calmodulin (CaM, 1:1000, Proteintech Group, Chicago, IL, USA) were incubated together and incubated overnight at 4 °C. After washing the PVDF membrane with Tris-buffered saline-Tween, the membrane was incubated with the following secondary antibodies: HRP-labeled goat anti-mouse IgG (1:10,000, Proteintech Group, Chicago, IL, USA) and HRP-labeled goat anti-rabbit IgG (1:10,000, Proteintech Group, Chicago, IL, USA). The membrane was washed using Tris-buffered saline-Tween. The immunoreactive bands were enhanced with a chemiluminescence solution (Biosharp, Beijing, China). The bands were imaged using a Protein Simple imager (ProteinSimple, San Jose, CA, USA), and the gray-scale values were analyzed using Image-Pro Plus (Media Cybernetics Inc., Warrendale, PA, USA).

### 2.10. Data Analysis

IBM’s Statistical Package for Social Sciences software (version 23.0; IBM Corp. Armonk, NY, USA) was used for statistical analysis, and data are expressed as mean ± standard error (X ± SEM). This study used an independent sample *t*-test to analyze the significance of differences in clinical background information, serum biochemical indicators, LFI, adipose tissue lipolysis, and inflammation-related protein abundance between healthy control cows and clinical ketosis cows. One-way analysis of variance and multiple comparisons were used to analyze the protein abundance of each group. Furthermore, Western blot results in this study were subjected to gray-scale analysis using Image-J software (Image J 1.8.0; National Institutes of Health, Bethesda, MD, USA). *p* < 0.05 was considered statistically significant, and *p* < 0.01 was considered highly statistically significant. Graphs were drawn using GraphPad Prism software (Prism 9.2.0; GraphPad Software, San Diego, CA, USA). * Denotes *p* < 0.05, and ** denotes *p* < 0.01.

## 3. Results

### 3.1. Characteristics and Blood Variables of Ketosis-Dairy Cows

Table 1 shows that cows in the CK exhibited lower milk production, glucose, and DMI (*p* < 0.05) and higher BCS and BW (*p* < 0.05) than those in the CON (Table 1). Additionally, cows in the CK exhibited significantly higher (*p* < 0.01) serum concentrations of BHB, NEFA, IL-6, IL-1β, and TNF-α than those in the CON. However, serum concentrations of the LFI were significantly lower (*p* < 0.01) among cows in the CK than among those in the CON.

### 3.2. The Abundance of CaM, Lipolysis, and Inflammation-Related Proteins in Dairy Cow Adipose Tissue

**Figure 1:**
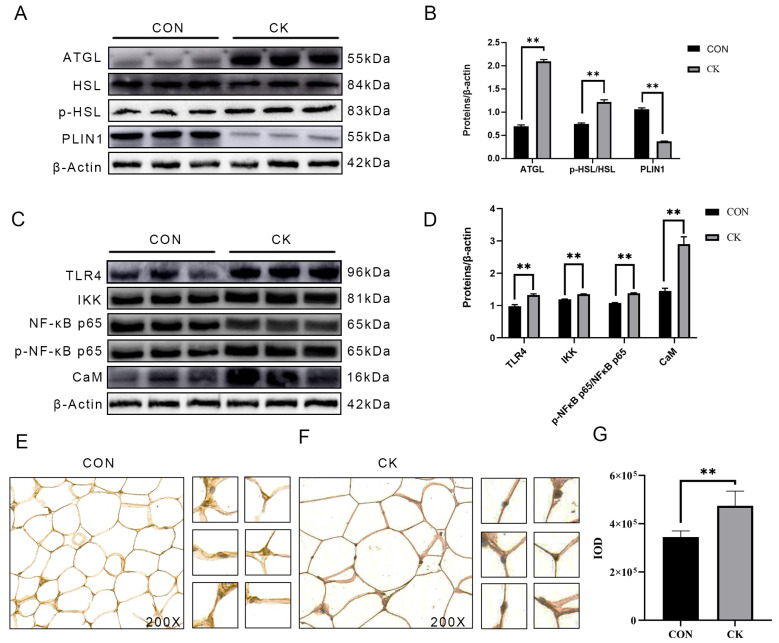
The abundance of ATGL, HSL, p-HSL, PLIN1, TLR4, IKK, p-NF-κB p65, NF-κB p65, and CaM in adipose tissue of healthy cows (*n* = 6) and cows with clinical ketosis (*n* = 6). CON = Control group, CK = Clinical ketosis group, ATGL = Adipose triacylglycerol lipase, HSL = Hormone-sensitive lipase, p-HSL = Phosphorylated hormone-sensitive lipase, PLIN1 = Perilipin-1, TLR4 = Toll-like receptor 4, IKK = Inhibitor of nuclear factor κB kinase subunit β, p-NF-κB p65 = Phosphorylated nuclear factor κB p65, NF-κB p65 = Nuclear factor κB p65, CaM = Calmodulin. (**A**) Representative Western blots of ATGL, HSL, p-HSL, and PLIN1. (**B**) Relative protein abundance of ATGL, p-HSL/HSL, and PLIN1. (**C**) Representative Western blots of TLR4, IKK, p-NF-κB p65, NF-κB p65, and CaM. (**D**) Relative protein abundance of TLR4, IKK, p-NF-κB p65/NF-κB p65, and CaM; (**E**) A picture under the microscope of adipose tissue from healthy dairy cows (200×). (**F**) A picture under the microscope of adipose tissue from clinical ketosis (200×). (**G**) Quantitative analysis of immunohistochemistry of CaM from adipose tissue of healthy and ketosis cows. This experiment was repeated thrice, and data are presented as mean ± SEM; ** *p* < 0.01.

Compared to cows in the CON, the protein abundance of ATGL and the phosphorylation of HSL was greater in adipose tissue of cows in the CK (*p* < 0.01, Figure 1A,B). However, the abundance of PLIN1 was lower among cows in the CK compared with those among cows in the CON (*p* < 0.01, Figure 1A,B). Protein abundance of inflammatory pathway (TLR4/IKK/NF-κB p65) proteins and CaM was greater among cows in the CK compared with cows in the CON (*p* < 0.01, Figure 1C,D).

### 3.3. Results of CaM Immunohistochemistry in Adipose Tissue of Dairy Cows

The abundance of CaM was increased in the adipose tissues of cows in the CK. Compared to those of cows in the CON, the adipocytes among cows in the CK showed hypertrophy and irregular cell morphology (*p* < 0.01, Figure 1E,G).

### 3.4. Effect of LPS Stimulation and CaM Silencing on the Abundance of Lipolysis-Related Proteins

**Figure 2:**
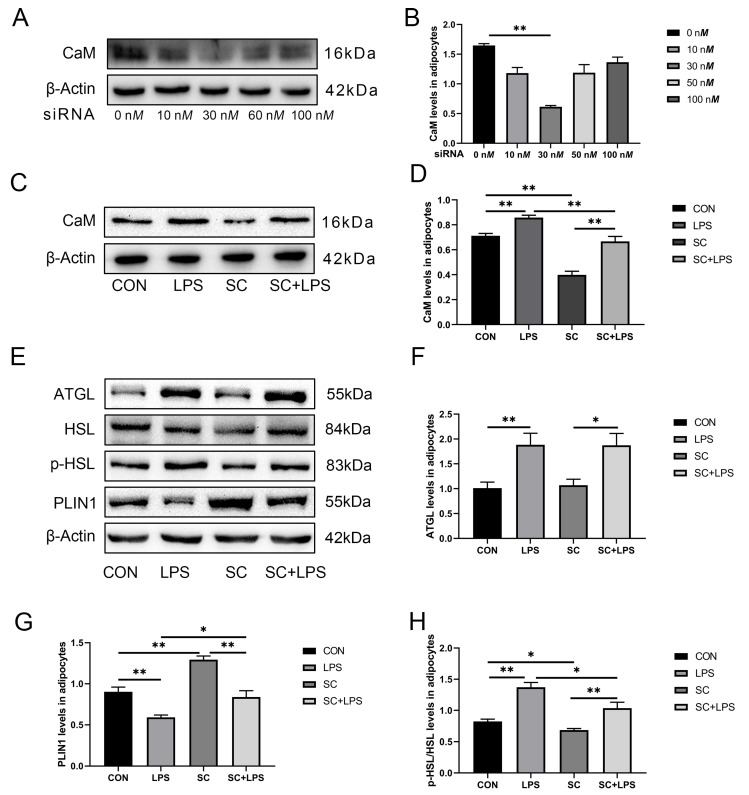
Effect of different siRNA concentrations on CaM abundance in adipocytes and the presence or absence of LPS on CaM, ATGL, HSL, p-HSL, and PLIN1 abundance in adipocytes. Adipocytes were transfected with CaM small interfering RNA (SC) for 48 h and cultured for 3 h in the presence or absence of LPS (4 μg/mL). CON = Control, LPS = Lipopolysaccharide treatment, SC = Calmodulin silencing, SC + LPS = Calmodulin silenced and lipopolysaccharide treatment, CaM = Calmodulin, ATGL = Adipose triacylglycerol lipase, HSL = Hormone-sensitive lipase, p-HSL = Phosphorylated hormone-sensitive lipase, PLIN1 = Perilipin-1. (**A**) Representative Western blots of CaM silenced by different siRNA concentrations. (**B**) Relative protein abundance of CaM after silencing CaM with varying concentrations of si-RNA. (**C**) Representative Western blots of CaM abundance in the case of silenced CaM and LPS stimulation. (**D**) Relative protein abundance of CaM. (**E**) Representative Western blots of ATGL, HSL, p-HSL, and PLIN1. (**F**) Relative protein abundance of ATGL. (**G**) Relative protein abundance of PLIN1. (**H**) Relative protein abundance of p-HSL/HSL. This experiment was repeated thrice, and data are presented as mean ± SEM: * *p* < 0.05, ** *p* < 0.01.

In this experiment, the addition of 30 nM siRNA was the most effective in downregulating CaM abundance (*p* < 0.01, Figure 2A,B).

Compared to cows in the CON, LPS treatment upregulated the abundance of CaM and ATGL and the phosphorylation of HSL but down-regulated the abundance of PLIN1 in cows in the CK (*p* < 0.01, Figure 2C–G). Silencing of CaM downregulated the abundance of CaM and the phosphorylation of HSL but upregulated the abundance of PLIN1 (*p* < 0.01, Figure 2C,D; *p* < 0.05, Figure 2E,H; *p* < 0.01, Figure 2E,G). Silencing of CaM could relieve the phosphorylation of HSL and the abundance of PLIN1 reduction by LPS when adipocytes were treated with LPS and CaM small interfering RNA simultaneously (*p* < 0.05, Figure 2E,H; *p* < 0.05, Figure 2E,G). However, silencing of CaM had no significant effect on ATGL (Figure 2E,F).

### 3.5. Effect of LPS Stimulation and CaM Silencing on the Abundance of Inflammation-Related Proteins

**Figure 3:**
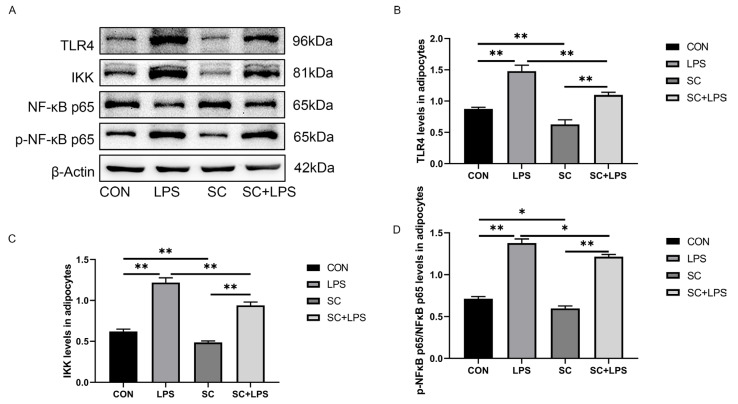
CaM silencing reduced LPS-induced inflammation-related proteins. Adipocytes were transfected with CaM small interfering RNA (SC) for 48 h and cultured for 3 h in the presence or absence of LPS (4 μg/mL). CON = Control, LPS = Lipopolysaccharide treatment, SC = Calmodulin silencing, SC + LPS = Calmodulin silenced and lipopolysaccharide treatment, TLR4 = Toll-like receptor 4, IKK = Inhibitor of nuclear factor κB kinase subunit β, p-NF-κB p65 = Phosphorylated nuclear factor κB p65, NF-κB p65 = Nuclear factor κB p65. (**A**) Representative Western blots of TLR4, IKK, p-NF-κB p65, and NF-κB p65. (**B**) Relative protein abundance of TLR4, IKK, p-NF-κB p65, and NF-κB p65. (**C**) Relative protein abundance of IKK. (**D**) Relative protein abundance of p-NF-κB p65/NF-κB p65. This experiment was repeated thrice, and data are presented as mean ± SEM; * *p* < 0.05, ** *p* < 0.01.

The abundance of TLR4 and IKK and p-NF-κB p65 was upregulated by LPS treatment (*p* < 0.01, Figure 3A,B; *p* < 0.01, Figure 3A,C; *p* < 0.01; Figure 3A,D) while silencing of CaM downregulated the abundance of TLR4 and IKK and p-NF-κB p65 (*p* < 0.01, Figure 3A,B; *p* < 0.01, Figure 3A,C; *p* < 0.05, Figure 3A,D) compared with the CON. Silencing of CaM could relieve the abundance of TLR4 and IKK and p-NF-κB p65 when adipocytes were treated with LPS and CaM small interfering RNA simultaneously (*p* < 0.01, Figure 3A,B; *p* < 0.01, Figure 3A,C; *p* < 0.05, Figure 3A,D).

### 3.6. Effect of LPS Stimulation and CaM Overexpression on the Abundance of Lipolysis-Related Proteins

**Figure 4:**
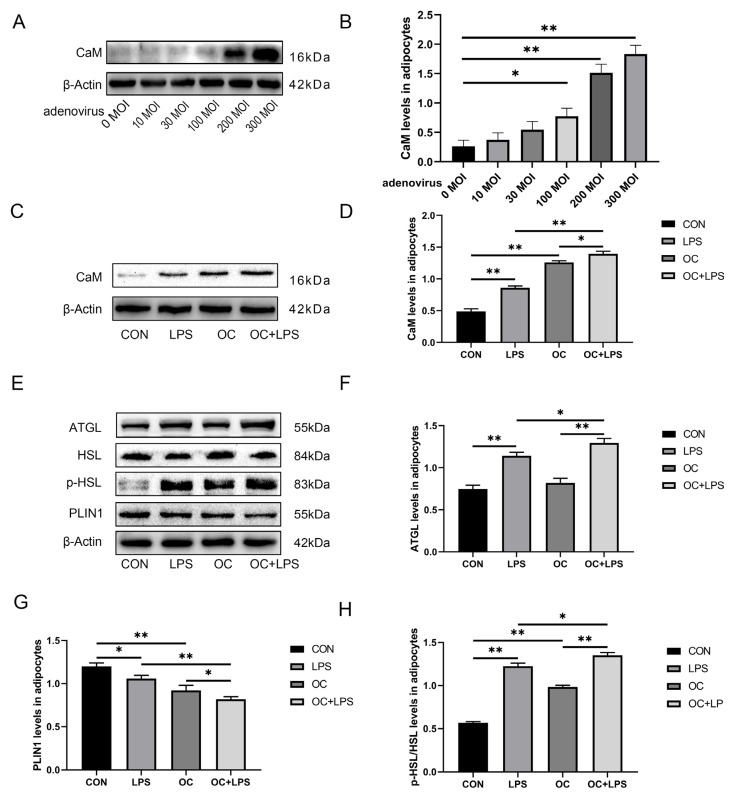
Effect of adenoviruses with different infection indices on CaM abundance. Adipocytes were transfected with OC-CaM (OC) for 48 h and cultured for 3 h in the presence or absence of LPS (4 μg/mL). CON = Control, LPS = Lipopolysaccharide treatment, OC = Calmodulin overexpression, OC + LPS = Calmodulin overexpression and lipopolysaccharide treatment, CaM = Calmodulin, ATGL = Adipose triacylglycerol lipase, HSL = Hormone-sensitive lipase, p-HSL = Phosphorylated hormone-sensitive lipase, PLIN1 = Perilipin-1. (**A**) Representative Western blots of CaM overexpression by different adenovirus concentrations. (**B**) After overexpression of CaM by adenovirus at various concentrations relative protein abundance of CaM. (**C**) Representative Western blots of CaM abundance under CaM overexpression and LPS stimulation conditions. (**D**) Relative protein abundance of CaM. (**E**) Representative Western blots of ATGL, HSL, p-HSL, and PLIN1. (**F**) Relative protein abundance of ATGL. (**G**) Relative protein abundance of PLIN1. (**H**) Relative protein abundance of p-HSL/HSL. This experiment was repeated thrice, and data are presented as mean ± SEM: * *p* < 0.05, ** *p* < 0.01.

In this experiment, CaM abundance was upregulated when the adenovirus reinfection index was 300 MOI (*p* < 0.01, Figure 4A,B), and notably, the addition of LPS modulated CaM abundance.

LPS treatment upregulated the abundance of CaM and ATGL and HSL phosphorylation (*p* < 0.01, Figure 4C,D; *p* < 0.01, Figure 4E,F; *p* < 0.01, Figure 4E,H) and downregulated PLIN1 abundance (*p* < 0.05, Figure 4E,G). Overexpression of CaM upregulated CaM abundance, HSL phosphorylation, and downregulated PLIN1 abundance (*p* < 0.01, Figure 4C,D; *p* < 0.01, Figure 4E,H; *p* < 0.01, Figure 4E,G) among cows in the CK than among those in the CON. Overexpression of CaM could enhance HSL phosphorylation. The abundance of ATGL increased by LPS (*p* < 0.05, Figure 4E,H; *p* < 0.05, Figure 4E,F), and overexpression of CaM promoted the abundance of PLIN1 decreased by LPS (*p* < 0.01, Figure 4E,G) when adipocytes were treated with LPS and adenovirus simultaneously.

### 3.7. Effect of LPS Stimulation and CaM Overexpression on the Abundance of Inflammation-Related Proteins

**Figure 5:**
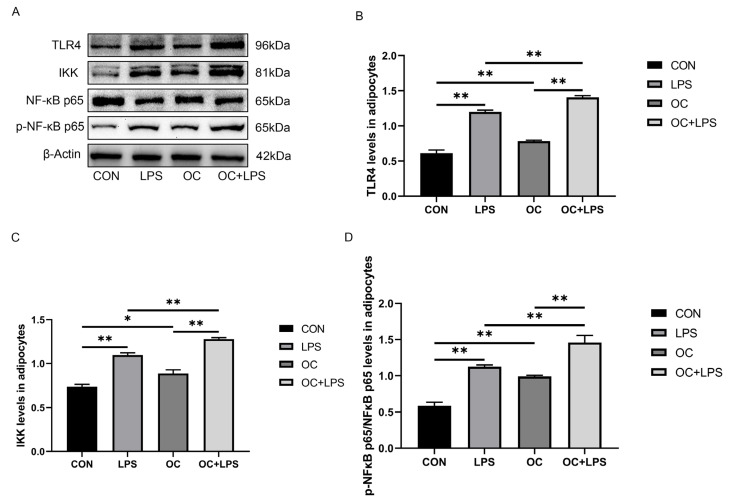
CaM overexpression upregulated LPS-induced abundance of inflammation-related proteins. Adipocytes were transfected with OC-CaM (OC) for 48 h and cultured for 3 h in the presence or absence of LPS (4 μg/mL). CON = Control, LPS = Lipopolysaccharide treatment, OC = Calmodulin overexpression, OC + LPS = Calmodulin overexpression and lipopolysaccharide treatment, TLR4 = Toll-like receptor 4, IKK = Inhibitor of nuclear factor κB kinase subunit β, p-NF-κB p65 = Phosphorylated nuclear factor κB p65, NF-κB p65 = Nuclear factor κB p65. (**A**) Representative Western blots of TLR4, IKK, p-NF-κB p65, and NF-κB p65. (**B**) Relative protein abundance of TLR4, IKK, p-NF-κB p65/NF-κB p65. (**C**) Relative protein abundance of IKK. (**D**) Relative protein abundance of p-NF-κB p65/NF-κB p65. This experiment was repeated thrice, and data are presented as mean ± SEM; * *p* < 0.05, ** *p* < 0.01.

LPS treatment upregulated the abundance of TLR4 and IKK and p-NF-κB p65 (*p* < 0.01, Figure 5A,B; *p* < 0.01, Figure 5A,C; *p* < 0.01, Figure 5A,D). Overexpression of CaM upregulated TLR4 and IKK abundance and NF-κB p65 phosphorylation (*p* < 0.01, Figure 5A,B; *p* < 0.05, Figure 5A,C; *p* < 0.01, Figure 5A,D) among cows in the CK than among those in the CON. Similarly, CaM overexpression could enhance the abundance of TLR4 and IKK and p-NF-κB p65 *(p* < 0.01, Figure 5A,B; *p* < 0.01, Figure 5A,C; *p* < 0.01, Figure 5A,D*)* when adipocytes were treated with LPS and adenovirus simultaneously.

## 4. Discussion

**Figure 6:**
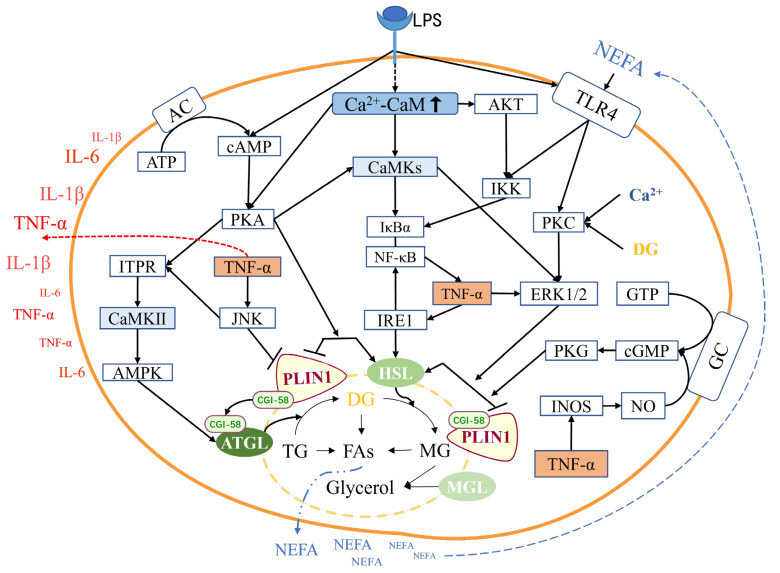
Regulatory network of CaM, lipolysis, and inflammatory pathway. Ca^2+^-CaM = Ca^2+^-calmodulin complex, LPS = lipopolysaccharide, CaMKII = Calmodulin kinase II, ATGL = Adipose triacylglycerol lipase, HSL = Hormone-sensitive lipase, PLIN1 = Perilipin-1, CGI-58 = Comparative gene identification-58, MGL = Monoacylglycerol lipase, TG = Triacylglycerol, DG = Diacylglycerol, MG = Monoacylglycerol, FAs = Fatty acids, NEFA = Non-esterified fatty acid, TLR4 = Toll-like receptor 4, IKK = Inhibitor of nuclear factor κB kinase subunit β, NF-κB = Nuclear factor κB, IκBα = NF-κB inhibitor-α, IL-6 = Interleukin-6, IL-1β = Interleukin-1β, TNF-α = Tumor necrosis factor-α, IRE1 = Inositol requires enzyme 1, ERK = Extracellular regulated protein kinases, PKC = Protein kinase C, GTP = Guanosine triphosphate, GC = Guanylate cyclase, cGMP = Cyclic guanosine monophosphate, PKG = Protein kinase G, INOS = Inducible nitric oxide synthase, NO = Nitric oxide, ATP = Adenosine triphosphate, AC = Adenylate cyclase, cAMP = Cyclic adenosine monophosphate, PKA = Protein kinase A, ITPR = 1,4,5-Inositol triphosphate receptor, JNK = C-Jun N-terminal kinase, MAPK = Mitogen activated protein kinase, AMPK = Adenosine monophosphate-activated protein kinase. Note: The white boxes are all derived from other references.

To better understand the relationship between CaM, lipolysis, and inflammation in adipocytes during clinical ketosis in dairy cows, we constructed a regulatory network of CaM based on the results of our experiment and the relevant literature on lipolysis and inflammation (Figure 6). The relationship between CaM, lipolysis, and inflammation is discussed as follows.

### 4.1. Relationship between CaM and Lipolysis in Adipocytes

CaM and its target protein CaMKII are essential in regulating adipocyte lipolysis metabolism (Figure 6). A previous study reported that CaM enhances adipocyte lipolytic metabolism via the cyclic adenosine monophosphate/protein kinase A/HSL pathway [30]. No report exists on the effect of CaM on ATGL and PLIN1, but CaM can bind to its target protein CaMKII to exert lipolysis. The Ca^2+^-CaMKII-ERK-HSL pathway can enhance HSL expression independent of the traditional protein kinase A pathway, stimulating lipolysis in 3T3-L1 preadipocytes. Furthermore, ATGL is positively regulated by CaMKII via the AMPK pathway, but the regulation of HSL by CaMKII is uncertain under different circumstances [31,32]. However, the transcription and expression of ATGL are controversial. Previous studies reported that ATGL mRNA transcription is upregulated by leptin in porcine adipocytes; however, its protein expression is downregulated [31,33]. Thus, the inconsistency between ATGL protein transcription and expression suggests posttranslational regulation of the enzyme. Alternatively, ATGL activity is regulated by transcription and, more importantly, comparative gene identification-58 and G0/G1 switch gene 2 [34,35].

Furthermore, lipolysis can be promoted by CaM-mediated inflammation. Figure 6 shows that PLIN1 was downregulated by TNF-α via p42/p44 and C-Jun N-terminal kinase [36]. Alternatively, HSL phosphorylation is induced by TNF-α via the nitric oxide/cyclic guanosine monophosphate pathway [37]. This study found no significant difference in ATGL expression between CaM silencing and overexpression conditions. However, upon LPS stimulation, the ATGL expression level was enhanced by CaM overexpression. In the presence or absence of LPS, the HSL phosphorylation was attenuated, and the expression of PLIN1 was enhanced under CaM silencing conditions, whereas the opposite was observed when CaM was overexpressed. Previous studies and our study consistently found that NEFA and BHB, as products of lipolysis, were upregulated in serum, CaM, and adipose tissue in cows with clinical ketosis, consistent with activation of the cell-tested pathway [38,39,40]. Therefore, our study suggests that in bovine adipocytes, lipolysis is promoted by CaM via enhancing p-HSL phosphorylation and attenuating PLIN1 expression; however, ATGL’s lipolysis is not mediated by CaM. It is unclear how LPS stimulation modifies the effect of CaM on ATGL; thus, more studies are required.

### 4.2. Relationship between CaM and Inflammation in Adipocytes

CaM can activate the TLR4/IKK/NF-κB pathway, and LPS can induce more intense inflammatory responses while increasing CaM abundance (Figure 6). LPS stimulation can activate the TLR4/IKK/NF-κB inflammatory pathway, leading to the upregulation of inflammatory cytokines, including TNF-α, IL-6, and IL-1β [41,42]. CaMKII is one of the essential target proteins of CaM [11] and is considered a key regulator of immunity and inflammation at different levels [14]. Moreover, TLR signaling requires interaction with the CaMKII pathway to be fully activated. In macrophages [43], T cells [44], and microglia [45], CaMKII can indirectly activate NF-κB via IκB or Akt, thereby promoting the production of proinflammatory cytokines, including TNF-α, IL-6, and type I interferon. Furthermore, CaMKII has a central role in regulating inflammation in myocardial infarction [46]. Our assay found that cellular expression of TLR4, IKK, p-NF-κB/NF-κB, and CaM was enhanced by LPS. When CaM was silenced, the expression of TLR4, IKK, and p-NF-κB/NF-κB was attenuated, and when CaM was overexpressed, opposite results were obtained. Furthermore, cows with clinical ketosis exhibited upregulation of TNF-α, IL-6, and IL-1β in their blood and increased CaM abundance in adipose tissue. These findings were consistent with the observed alterations in inflammatory pathways and CaM abundance in vitro. Therefore, our study demonstrated that LPS could enhance CaM expression, and CaM and LPS synergistically activated the TLR4/IKK/NF-κB inflammatory pathway in adipocytes. However, further investigation is required to elucidate the specific molecular mechanisms underlying the upregulation of CaM by LPS; the specific pathways by which LPS upregulates CaM require more investigation.

### 4.3. Relationship between Inflammation and Lipolysis in Adipocytes

The inflammatory response within adipocytes can induce lipolysis, and excessive lipolysis may further exacerbate the inflammatory response (Figure 6). LPS-induced inflammation upregulates ATGL expression and stimulates HSL phosphorylation. LPS-induced inflammation increases ATGL and HSL expression, promoting HSL phosphorylation [41,42]. Rat ATGL can be directly activated by LPS and its core component, lipid A [42]. When cells are exposed to LPS, ATGL is activated, followed by HSL activation. Therefore, the breakdown of triacylglycerol by ATGL is considered part of the acute phase response to endotoxemia, and whole-body energy expenditure is increased [27]. Furthermore, studies have shown that LPS can mediate lipolysis by activating the IKK/NF-κB inflammatory pathway and cyclic adenosine monophosphate/protein kinase A/HSL classical lipolysis pathway [47]. Inflammation can also enhance adipocyte lipolysis and reduce insulin sensitivity via multiple pathways, including TLR4/ERK1/2/HSL [42], inositol-requiring enzyme/HSL [48], TNF-α/nitric oxide synthase/nitric oxide/cyclic guanosine monophosphate [37], TNF-α/GTP-binding protein/inhibitory adenylate cyclase g protein [49], TNF-α/p42/p44, and c-Jun N-terminal kinase/PLIN1 [36]. These pathways subsequently induce HSL/PLIN phosphorylation [27]. Studies have demonstrated that inflammation can activate lipolysis via the c-Jun N-terminal kinase-1,4,5-inositol triphosphate receptor-CaM/CaMKII pathway and contribute to the positive feedback loop between inflammatory and metabolic signals in obesity [12]. Low levels of PLIN1, which implies stronger lipolysis, promote NF-κB pathway activation and inflammatory cytokine synthesis, including TNF-α, IL-1β, and IL-6, and increase fatty acid and triacylglycerol synthesis [26].

Moreover, NEFA produced by excessive lipolysis can directly act on TLR4 or IKK, further activating the NF-κB pathway and aggravating the inflammatory response [50]. Our assay found that the abundance of CaM, ATGL, and p-HSL was significantly enhanced in adipocytes after LPS stimulation, while the expression of PLIN1 was significantly attenuated, indicating enhanced lipolysis. Furthermore, adipose tissue lipolysis was enhanced in cows with clinical ketosis, as characterized by the increased serum levels of NEFA and BHB. Simultaneously, the TLR4/IKK/NF-κB pathway in adipose tissue was activated, and the levels of TNF-α, IL-1β, and IL-6 in serum were upregulated. Therefore, our study demonstrated that LPS-induced inflammation could enhance lipolysis via multiple pathways on ATGL, HSL, and PLIN lipolysis-related proteins, while NEFA released from lipolysis could act on TLR4 further to activate TLR4/IKK/NF-κB pathway-mediated inflammation.

**Figure 7:**
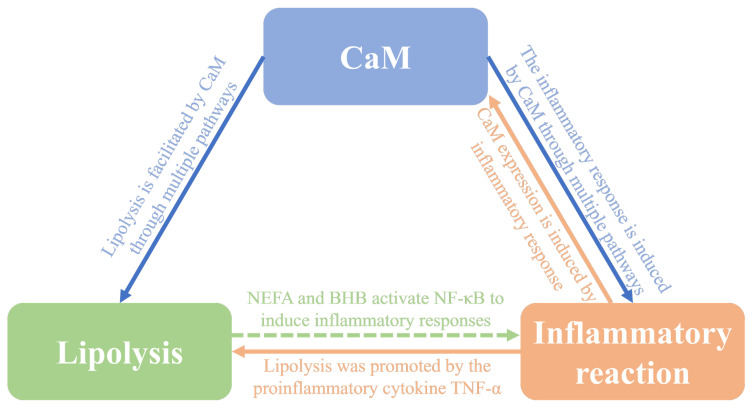
Positive feedback loops for CaM, inflammation, and lipolytic signaling. CaM = Calmodulin, NEFA = Non-esterified fatty acid, NF-κB = Nuclear factor κB, TNF-α = Tumor necrosis factor-α.

Figure 7 displays that inflammatory cytokines released upon activation of NF-κB, such as TNF-α, could mediate lipolysis and upregulate the abundance of CaM through ATGL, HSL, and PLIN1. NEFA and BHB released from excessive lipolysis aggravate the inflammatory response, creating a positive feedback loop of CaM, inflammation, and lipolysis signals.

## 5. Conclusions

We observed cows with clinical ketosis in a state of negative energy balance, increased lipolysis, liver damage, and inflammation. This state is associated with increased expression of CaM; activation of the TLR4/IKK/NF-κB inflammatory pathway; and increased lipolysis of ATGL, p-HSL/HSL, and PINL1 in adipose tissue. We confirmed that CaM positively regulates lipolysis and inflammation in adipocytes. These findings imply that CaM may play an essential role in the pathogenesis of clinical ketosis in dairy cows. Reducing CaM abundance could help control the inflammatory response in the adipose tissue of dairy cows during the development of clinical ketosis.

## Figures and Tables

**Table 1:** Characteristics and blood variables of selected dairy cows ^1^.

Item	CON (*n* = 6)	CK (*n* = 6)	*p*-Value
Milk production (L/day)	38.34 ± 0.27	27.50 ± 0.21	<0.001
DMI (kg/day)	21.32 ± 0.47	19.75 ± 0.40	0.028
BW (kg)	613.29 ± 6.24	643.70 ± 7.18	0.010
BCS	2.67 ± 0.17	3.32 ± 0.22	0.041
Glucose (mmol/L)	4.03 ± 0.07	2.23 ± 0.03	<0.001
BHB (mmol/L)	0.38 ± 0.05	3.29 ± 0.11	<0.001
NEFA (mmol/L)	0.32 ± 0.06	1.18 ± 0.11	<0.001
AST (U/L)	72.00 ± 1.59	156.40 ± 3.52	<0.001
ALT (U/L)	18.80 ± 1.18	35.53 ± 1.87	<0.001
LFI	1.08 ± 0.86	−7.53 ± 1.24	<0.001
IL-6 (ng/L)	0.46 ± 0.02	1.26 ± 0.05	<0.001
IL-1β (ng/L)	1.50 ± 0.31	6.08 ± 0.59	<0.001
TNF-α (ng/L)	69.24 ± 3.95	102.49 ± 4.29	<0.001

^1^ Data were analyzed using the *t*-test. Data are presented as median and interquartile range. CON = Control group, CK = Clinical ketosis group, BW = Body weight, DMI = Dry matter intake, BCS = Body condition score, AST = Aspartate aminotransferase, ALT = Alanine transaminase, LFI = Liver function index. IL-6 = Interleukin-6, IL-1β = Interleukin-1β, TNF-α = Tumor necrosis factor-α. Data are presented as mean ± SEM.

## Data Availability

The data that support the findings of this study are available from the corresponding author upon reasonable request.

## Supplementary Materials

The following supporting information can be downloaded at: https://www.mdpi.com/article/10.3390/ani14111678/s1, Table S1: Ingredient and nutrient composition of the diets.

## References

[1] Grummer R.R. (1995). Impact of changes in organic nutrient metabolism on feeding the transition dairy cow. J. Anim. Sci..

[2] Loor J.J., Everts R.E., Bionaz M., Dann H.M., Morin D.E., Oliveira R., Rodriguez-Zas S.L., Drackley J.K., Lewin H.A. (2007). Nutrition-induced ketosis alters metabolic and signaling gene networks in liver of periparturient dairy cows. Physiol. Genom..

[3] Contreras G.A., Kabara E., Brester J., Neuder L., Kiupel M. (2015). Macrophage infiltration in the omental and subcutaneous adipose tissues of dairy cows with displaced abomasum. J. Dairy Sci..

[4] Chirivi M., Cortes-Beltran D., Munsterman A., O’Connor A., Contreras G.A. (2023). Lipolysis inhibition as a treatment of clinical ketosis in dairy cows: A randomized clinical trial. J. Dairy Sci..

[5] Schweiger M., Schreiber R., Haemmerle G., Lass A., Fledelius C., Jacobsen P., Tornqvist H., Zechner R., Zimmermann R. (2006). Adipose Triglyceride Lipase and Hormone-sensitive Lipase Are the Major Enzymes in Adipose Tissue Triacylglycerol Catabolism. J. Biol. Chem..

[6] Gruber A., Cornaciu I., Lass A., Schweiger M., Poeschl M., Eder C., Kumari M., Schoiswohl G., Wolinski H., Kohlwein S.D. (2010). The N-terminal region of comparative gene identification-58 (CGI-58) is important for lipid droplet binding and activation of adipose triglyceride lipase. J. Biol. Chem..

[7] Brasaemle D.L., Rubin B., Harten I.A., Gruia-Gray J., Kimmel A.R., Londos C. (2000). Perilipin A increases triacylglycerol storage by decreasing the rate of triacylglycerol hydrolysis. J. Biol. Chem..

[8] Bertoni G., Trevisi E. (2013). Use of the liver activity index and other metabolic variables in the assessment of metabolic health in dairy herds. Vet. Clin. N. Am. Food Anim. Pract..

[9] Cardoso F.C., LeBlanc S.J., Murphy M.R., Drackley J.K. (2013). Prepartum nutritional strategy affects reproductive performance in dairy cows. J. Dairy Sci..

[10] Tian H., Liu C., Zou X., Wu W., Zhang C., Yuan D. (2015). MiRNA-194 Regulates Palmitic Acid-Induced Toll-Like Receptor 4 Inflammatory Responses in THP-1 Cells. Nutrients.

[11] Berchtold M.W., Antonio V. (2014). The many faces of calmodulin in cell proliferation, programmed cell death, autophagy, and cancer. Biochim. Biophys. Acta BBA Mol. Cell Res..

[12] Dai W., Choubey M., Patel S., Singer H., Ozcan L. (2021). Adipocyte CAMK2 deficiency improves obesity-associated glucose intolerance. Mol. Metab..

[13] Huang S.H., Shen W.J., Yeo H.L., Wang S.M. (2010). Signaling pathway of magnolol-stimulated lipolysis in sterol ester-loaded 3T3-L1 preadipocyes. J. Cell. Biochem..

[14] Lin M.Y., Zal T., Ch’En I.L., Gascoigne N.R.J., Hedrick S.M. (2005). A Pivotal Role for the Multifunctional Calcium/Calmodulin-Dependent Protein Kinase II in T Cells: From Activation to Unresponsiveness. J. Immunol..

[15] Hughes K., Edin S., Antonsson A., Grundstrm T. (2001). Calmodulin-dependent kinase II mediates T cell receptor/CD3- and phorbol ester-induced activation of IkappaB kinase. J. Biol. Chem..

[16] Pereira C., Schaer D.J., Bachli E.B., Kurrer M.O., Schoedon G. (2008). Wnt5A/CaMKII Signaling Contributes to the Inflammatory Response of Macrophages and Is a Target for the Antiinflammatory Action of Activated Protein C and Interleukin-10. Arterioscler. Thromb. Vasc. Biol..

[17] Mével E., Shutter J.A., Ding X., Mattingly B.T., Williams J.N., Li Y., Huls A., Kambrath A.V., Trippel S.B., Wagner D. (2022). Systemic inhibition or global deletion of CaMKK2 protects against post-traumatic osteoarthritis. Osteoarthr. Cartil..

[18] Wang N., Wang J., Zhang Y., Zeng Y., Hu S., Bai H., Hou Y., Wang C., He H., He L. (2021). Imperatorin ameliorates mast cell-mediated allergic airway inflammation by inhibiting MRGPRX2 and CamKII/ERK signaling pathway. Biochem. Pharmacol..

[19] Xu Q., Li X., Ma L., Loor J.J., Li X. (2019). Adipose tissue proteomic analysis in ketotic or healthy Holstein cows in early lactation1. J. Anim. Sci..

[20] Percie du Sert N., Hurst V., Ahluwalia A., Alam S., Avey M.T., Baker M., Browne W.J., Clark A., Cuthill I.C., Dirnagl U. (2020). The ARRIVE guidelines 2.0: Updated guidelines for reporting animal research. PLoS Biol..

[21] Ferguson J.D., Galligan D.T., Thomsen N. (1994). Principal Descriptors of Body Condition Score in Holstein Cows. J. Dairy Sci..

[22] Vanholder T., Papen J., Bemers R., Vertenten G., Berge A.C.B. (2015). Risk factors for subclinical and clinical ketosis and association with production parameters in dairy cows in the Netherlands. J. Dairy Sci..

[23] Wang Z., Song Y., Zhang F., Zhao C., Fu S., Xia C., Bai Y. (2022). Early warning for inactive ovaries based on liver function index, serum MDA, IL-6, FGF21 and ANGPTL8 in dairy cows. Ital. J. Anim. Sci..

[24] Xu Q., Fan Y., Loor J.J., Liang Y., Sun X., Jia H., Zhao C., Xu C. (2021). Adenosine 5′-monophosphate-activated protein kinase ameliorates bovine adipocyte oxidative stress by inducing antioxidant responses and autophagy. J. Dairy Sci..

[25] Wang L., Johnson J.A., Chang D.W., Zhang Q. (2013). Decellularized musculofascial extracellular matrix for tissue engineering. Biomaterials.

[26] Zhang S., Liu G., Xu C., Liu L., Zhang Q., Xu Q., Jia H., Li X., Li X. (2018). Perilipin 1 Mediates Lipid Metabolism Homeostasis and Inhibits Inflammatory Cytokine Synthesis in Bovine Adipocytes. Front. Immunol..

[27] Xu Q., Jia H., Ma L., Liu G., Xu C., Li Y., Li X., Li X. (2019). All-trans retinoic acid inhibits lipopolysaccharide-induced inflammatory responses in bovine adipocytes via TGFβ1/Smad3 signaling pathway. BMC Vet. Res..

[28] Xu Q., Fan Y., Loor J.J., Jiang Q., Zheng X., Wang Z., Yang T., Sun X., Jia H., Li X. (2022). Effects of diacylglycerol O-acyltransferase 1 (DGAT1) on endoplasmic reticulum stress and inflammatory responses in adipose tissue of ketotic dairy cows. J. Dairy Sci..

[29] Sun X., Li X., Jia H., Loor J.J., Bucktrout R., Xu Q., Wang Y., Shu X., Dong J., Zuo R. (2019). Effect of heat-shock protein B7 on oxidative stress in adipocytes from preruminant calves. J. Dairy Sci..

[30] Kawai A. (1985). The role of calmodulin in hormone-stimulated lipolysis. Metabolism.

[31] Roepstorff C., Vistisen B., Donsmark M., Nielsen J.N., Galbo H., Green K.A., Hardie D.G., Wojtaszewski J.F., Richter E.A., Kiens B. (2004). Regulation of hormone-sensitive lipase activity and Ser563 and Ser565 phosphorylation in human skeletal muscle during exercise. J. Physiol..

[32] Kim S.J., Tang T., Abbott M., Viscarra J.A., Wang Y., Sul H.S. (2016). AMPK Phosphorylates Desnutrin/ATGL and Hormone-Sensitive Lipase To Regulate Lipolysis and Fatty Acid Oxidation within Adipose Tissue. Mol. Cell. Biol..

[33] Li Y.C., Zheng X.L., Liu B.T., Yang G.S. (2010). Regulation of ATGL expression mediated by leptin in vitro in porcine adipocyte lipolysis. Mol. Cell. Biochem..

[34] Yamaguchi T. (2010). Crucial Role of CGI-58/α/β Hydrolase Domain-Containing Protein 5 in Lipid Metabolism. Biol. Pharm. Bull..

[35] Yang X., Lu X., Lombès M., Rha G.B., Chi Y.I., Guerin T.M., Smart E.J., Liu J. (2010). The G0/G1 Switch Gene 2 Regulates Adipose Lipolysis through Association with Adipose Triglyceride Lipase. Cell Metab..

[36] Rydén M., Arvidsson E., Blomqvist L., Perbeck L., Dicker A., Arner P. (2004). Targets for TNF-alpha-induced lipolysis in human adipocytes. Biochem. Biophys. Res. Commun..

[37] Lien C.C., Au L.C., Tsai Y.L., Ho L.T., Juan C.C. (2009). Short-term regulation of tumor necrosis factor-alpha-induced lipolysis in 3T3-L1 adipocytes is mediated through the inducible nitric oxide synthase/nitric oxide-dependent pathway. Endocrinology.

[38] Dervishi E., Plastow G., Hoff B., Colazo M. (2021). Common and specific mineral and metabolic features in dairy cows with clinical metritis, hypocalcaemia or ketosis. Res. Vet. Sci..

[39] Shin E.K., Jeong J.K., Choi I.S., Kang H.G., Hur T.Y., Jung Y.H., Kim I.H. (2015). Relationships among ketosis, serum metabolites, body condition, and reproductive outcomes in dairy cows. Theriogenology.

[40] Kerwin A.L., Burhans W.S., Mann S., Nydam D.V., Wall S.K., Schoenberg K.M., Perfield K.L., Overton T.R. (2022). Transition cow nutrition and management strategies of dairy herds in the northeastern United States: Part II-Associations of metabolic- and inflammation-related analytes with health, milk yield, and reproduction. J. Dairy Sci..

[41] Chirivi M., Rendon C., Myers M., Prom C., Roy S., Sen A., Lock A., Contreras G. (2021). Lipopolysaccharide induces lipolysis and insulin resistance in adipose tissue from dairy cows. J. Dairy Sci..

[42] Zu L., He J., Jiang H., Xu C., Pu S., Xu G. (2009). Bacterial Endotoxin Stimulates Adipose Lipolysis via Toll-Like Receptor 4 and Extracellular Signal-regulated Kinase Pathway. J. Biol. Chem..

[43] Liu X., Yao M., Li N., Wang C., Zheng Y., Cao X. (2008). CaMKII promotes TLR-triggered proinflammatory cytokine and type I interferon production by directly binding and activating TAK1 and IRF3 in macrophages. Blood.

[44] Singh M.V., Swaminathan P.D., Luczak E.D., Kutschke W., Weiss R.M., Anderson M.E. (2012). MyD88 mediated inflammatory signaling leads to CaMKII oxidation, cardiac hypertrophy and death after myocardial infarction. J. Mol. Cell. Cardiol..

[45] Jeon S., Kim S.H., Shin S.Y., Lee Y.H. (2018). Clozapine reduces Toll-like receptor 4/NF-κB-mediated inflammatory responses through inhibition of calcium/calmodulin-dependent Akt activation in microglia. Prog. Neuropsychopharmacol. Biol. Psychiatry.

[46] Rusciano M.R., Sommariva E., Douin-Echinard V., Ciccarelli M., Poggio P., Maione A.S. (2019). CaMKII Activity in the Inflammatory Response of Cardiac Diseases. Int. J. Mol. Sci..

[47] Grisouard J., Bouillet E., Timper K., Radimerski T., Dembinski K., Frey D.M., Peterli R., Zulewski H., Keller U., Müller B. (2012). Both inflammatory and classical lipolytic pathways are involved in lipopolysaccharide-induced lipolysis in human adipocytes. Innate Immun..

[48] Foley K.P., Chen Y., Barra N.G., Heal M., Schertzer J.D. (2021). Inflammation promotes adipocyte lipolysis via IRE1 kinase. J. Biol. Chem..

[49] Gasic S., Tian B., Green A. (1999). Tumor necrosis factor alpha stimulates lipolysis in adipocytes by decreasing Gi protein concentrations. J. Biol. Chem..

[50] Wang N., Chen X., Ji Y., Lan T., Yan W., Xu Y., Gong G. (2023). Anti-inflammatory Effect of a Limonin Derivative In Vivo and Its Mechanisms in RAW264.7 Cells. Inflammation.

