# Supplementary material for: Calmodulin Contributes to Lipolysis and Inflammatory Responses in Clinical Ketosis Cows through the TLR4/IKK/NF-κB Pathway
Source: Animals (Basel). 2024 Jun 4;14(11):1678. doi: 10.3390/ani14111678 (PMC11171032; doi:10.3390/ani14111678)
Supplement: Supplementary file 1 [file animals-14-01678-s001.zip › Supplemental Table S1.pdf]

Supplemental Table S1. Ingredient and nutrient composition of the diets.

**Item (% of DM)**	
Corn silage	40.00
Corn	35.00
Wheat bran	8.00
Soybean meal	5.00
Sunflower	8.00
NaCl	1.00
Premix*	1.80
$NaHCO_3$	1.20
Total	100.00
Nutrient composition (% of DM)	
NEL (MJ/Kg)	6.70
CP	15.20
NDF	33.45
ADF	17.20
NFC	40.40
Ca	0.70
P	0.50

*The premix provided the following per kg of diets: VA 200,000 IU, VD 70,000 IU, VE 1,000 IU, Fe (salts) 2,000 mg, Cu (salts) 600 mg, Zn (salts) 2,400 mg, Mn (salts) 1,300 mg, I 6 mg, Co (salts) 7 mg.

DM, Dry Matter; NEL, Net energy for lactation; CP, Crude protein; NDF, Neutral detergent fibre; ADF, Acid detergent fibre; NFC, Non-fibre carbohydrate. VA, vitamin A; VE, Vitamin E; VD, Vitamin D.
